# Academic metrics and publication history of presenters at the American Shoulder and Elbow Surgeons closed annual conferences from 2016 to 2022

**DOI:** 10.1016/j.xrrt.2025.01.013

**Published:** 2025-04-09

**Authors:** Aaron M. Atlas, Christina Montesano, Brandon Klein, John Grossi, Kyle Cullin, Randy M. Cohn

**Affiliations:** a Department of Orthopedic Surgery, Northwell, New Hyde Park, NY, USA; b Department of Orthopedic Surgery at Huntington Hospital, Huntington, NY, USA; c Department of Orthopedic Surgery, Zucker School of Medicine at Hofstra/Northwell, Hempstead, NY, USA; d New York Institute of Technology College of Osteopathic Medicine, Old Westbury, NY, USA; e Lake Erie College of Osteopathic Medicine – Bradenton Campus, Bradenton, FL, USA

**Keywords:** Academic metrics, ASES conference, Shoulder and elbow surgeons, Publication history, Shoulder and Elbow Surgery, Education, Conference Presenter Metrics

## Abstract

**Background:**

The American Shoulder and Elbow Surgeons (ASES) holds annual educational meetings to highlight new advances in the field of shoulder and elbow surgery. This study aims to assess the academic metrics of lecturers, ICL instructors, and moderators at ASES annual meetings.

**Methods:**

A review of selected presenters (lecturers, ICL instructors, and moderators) at ASES annual meetings from 2016 to 2022 was performed. Presentation topics were categorized into ten topic-specific content areas. Presenters' Hirsch index (H-index), number of topic-specific publications, and field-weighted citation impact (FWCI) were examined to assess academic metrics.

**Results:**

Overall, 348 presenters were evaluated. 76.6% of presentations were provided by physicians with at least 1 prior topic-specific publication. Lecturers and symposium moderators had the highest average proportion of presenters with previous topic-specific publications (81.5%), followed by ICL instructors (72.7%). The most commonly presented content topics included arthroplasty techniques (16.8%), followed by rotator cuff tears (15.9%) and surgical complications (12.4%). Infections-prophylaxis and treatment was the least presented topic (3.1%). Lecturers discussing infection held the highest average FWCI (2.55), while elbow injuries had the highest average H-index (59.6). ICL instructors discussing rotator cuffs had the highest average FWCI (4.7), while ICL instructors on biologics had the highest H-index (58.0). Symposium moderators on infection held the highest average FWCI (2.8), while those discussing nonclinical/orthopedic topics had the highest average H-index (55).

**Conclusion:**

A high percentage of ASES presenters had previous topic-specific publications as well as distinguishable H-indices and FWCI. Speakers at ASES annual meetings are academically accomplished within the field of shoulder and elbow surgery.

Annual surgical society meetings provide a medium that allows for the presentation and evaluation of the latest data, technology, and surgical techniques within highly specialized surgical fields.^18^ The American Shoulder and Elbow Surgeons (ASES) holds annual closed (members only) educational meetings in the fall to highlight new advances in surgical techniques, research and recommendations on patient care relating to the shoulder and elbow.^3^

ASES closed annual conferences provide a variety of events including lectures, instructional course lectures (ICLs), and clinical symposium overseen by moderators.^17^ With national and international exposure, presented information may be incorporated by attendees into their clinical practice. Owing to the impact of these presentations, speakers must be chosen carefully to ensure the most current information and recommendations are being presented. Conference planning committees have many factors to consider when selecting presenters, including their academic experience, previous research publications, clinical prowess, public speaking history, and perspectives on relevant surgical controversies. As publication history and academic metrics may represent a surgeon's contribution to emerging orthopedic literature, speakers with extensive academic backgrounds may be well informed on the particular topics of interest.

Research is limited on assessing the academic metrics of lecturers, ICL instructors, and moderators at ASES annual meetings. Authors sought to answer the following questions: (1) Is there a focus on specific content areas in which presenters have an abundance of previous topic-specific publications? (2) Do academic metrics differ based on various presenter roles? (3) How do ASES presenters compare to other national orthopedic subspecialty society speakers?

## Methods

Programs of 2016-2022 ASES closed annual meetings were obtained from a society representative via email correspondence. The organization of individual conference programs varied annually, with events including: lectures, ICLs, symposiums, scientific poster presentations, paper abstract presentations, anatomic case presentations, industry-sponsored workshops, and live surgery sessions. Due to the variation of events, we chose to focus on selected presenter events – lectures, ICLs, and symposia to assess, as each of these events was included in the majority of the annual conferences programs. For the purposes of this paper, we described symposia as an event with moderators that oversaw a discussion. We described lectures as an event where a presentation included 1 or more physicians and there were no moderators; these lectures encompassed invited talks on specific topics only. ICLs were described as an event labeled as an ICL. All conference programs were reviewed by two authors to determine inclusion of individual event presentations. In the event of disagreement between the two reviewers, a third observer was consulted to reach a consensus. There was annual variability in the frequency of presentation-type reviewers selected lectures, ICLs, and symposiums from each year despite this annual fluctuation. When presentations were led by two or more physicians, each moderator was evaluated independently. Full-text abstracts and associated published manuscripts were thoroughly reviewed to determine whether they were topic-specific. Manuscripts were deemed topic-specific if the paper's focus was in reference to the content category of the speaker's presentation. If a paper was deemed nontopic-specific, it was not included in the presenter's total publication count. A minimum threshold of 1 publication was used to include presenters with previous topic-specific publications. Industry-sponsored workshops were excluded to focus on those presenting orthopedic literature relating to the shoulder and elbow.

### Presenter-specific variables

For each included presentation, corresponding presenter names and degrees, type of presentation, and topic of presentation were recorded from the annual conference programs. The presenter role (lecturer, ICL instructor, or symposium moderator) was also recorded as listed in the conference program. Presenters' names, gender, type of presentation (lecture, ICL, or symposium), presentation name, Hirsch index (H-index), number of topic-specific publications, and field-weighted citation impact (FWCI) were recorded in Microsoft Excel (Microsoft Corp., Redmond, WA, USA). If a presenter appeared multiple times, each event they presented on was counted separately for the purposes of this study. Previous peer-reviewed publications were identified by searching the speaker's full name in the Scopus and PubMed databases. The full text manuscripts of previous publications were reviewed to ensure that its topic pertained to the presenter's ASES conference presentation. Manuscripts which were published after the date of presentation were not included. To evaluate peer-reviewed publications, authorship in textbook chapters, case reports, expert opinions, and instructional case lectures were excluded. The FWCI of each peer-reviewed paper and H-index for each author were recorded as listed on the SCOPUS database.^15^^,^^14^

### Topic-specific variables

To assess academic metrics of lecturers by content topic, authors created the following ten topic-specific content areas: arthroplasty techniques, biologics, elbow injuries, fractures/stiffness, infections, rotator cuff tears (RCTs), shoulder instability, soft tissue injuries, surgical complications, and nonclinical/nonorthopedic. Nonclinical/nonorthopedic presentations were evaluated based on the presenter's H-index only (FWCI not assessed). These categories were developed by authors based on previous ASES conference program categorization and. analysis of similar literature which reported on presenters from other orthopedic surgical societies.^16^

### Statistical analysis

Speaker demographics and publication data were calculated as counts, averages and percentages. The FWCI and H-index of presenters was described using means and standard deviations where appropriate, and were stratified by content category and by presentation type. The academic metrics and publication history of speakers were compared using descriptive variables.

## Results

There were a total of 348 presenters evaluated across seven annual meetings (2016-2022) (Table I). 328 (94.3%) presenters were male 20 (5.7%) were female. The majority of these presenters were lecturers (52.2%), followed by symposium moderators (24.8%) and ICL instructors (23.0%) (Table II).

**Table I:** Presenter and presentation details.

Conference year	Total (n)	Male	Female
2016	17	16 (94.1%)	1 (5.9%)
2017	47	47 (100%)	0
2018	114	108 (94.7%)	6 (5.3%)
2019	67	67 (100%)	0
2020	18	18 (100%)	0
2021	52	46 (88.5%)	6 (11.5)
2022	33	26 (78.8%)	7 (21.2%)
Total	348	328	20

Presentation type	Number of presentations		
Lectures	118 (52.2%)		
Instructional course lectures	52 (23.0%)		
Moderators	56 (24.8%)		
Total	226

**Table II:** Presentation frequency by topic.

Category	Conference presentation frequency	Presenters with a related publication
Arthroplasty techniques	38 (16.8%)	74.2% (49/66)
Biologics	11 (4.87%)	61.5% (8/13)
Elbow injury	25 (11.1%)	83.0% (39/47)
Fracture/stiffness	23 (10.2%)	78.8% (27/33)
Infection	7 (3.10%)	77.8% (7/9)
Rotator cuff tear	36 (15.9%)	90.2% (46/51)
Shoulder instability	26 (11.5%)	74.5% (35/47)
Soft tissue injury	10 (4.42%)	65.2% (15/23)
Surgical complications	28 (12.4%)	61.3% (19/31)
Nonclinical	22 (9.73%)	-
Total	226	-

There were a total of 226 presentations across seven annual meetings (2016-2022). The majority of presenters (76.6%) had at least 1 prior topic-specific publication. The most commonly presented content topics included arthroplasty techniques (16.8%), followed by RCT (15.9%) and surgical complications (12.4%). Infections-prophylaxis and treatment was the least presented topic (3.1%) (Table III).

**Table III:** Academic metrics of lecturers.

Category	Number of lectures	Lecturers with prior topic-specific publication	Average FWCI	Average H-index
Arthroplasty techniques	19	18/24 (75%)	2.0	44.0
Biologics	6	7/7 (100%)	2.4	39.6
Elbow injury	8	9/12 (75.0%)	1.4	59.6
Fracture/stiffness	9	9/9 (100%)	2.3	48.1
Infection	5	4/6 (66.7%)	2.6	38.3
Rotator cuff tear	23	23/27 (85.2%)	2.4	36.7
Shoulder instability	11	14/15 (93.3%)	1.9	47.0
Soft tissue injury	2	2/2 (100%)	1.6	29.5
Surgical complications	21	15/22 (68.2%)	1.6	44.3
Nonclinical	14	-	-	42.0
Total	118	101/124 (81.5%)	2.0	42.9

*FWCI*, field-weighted citation impact; *H-index*, Hirsch index.

**Figure 1:**
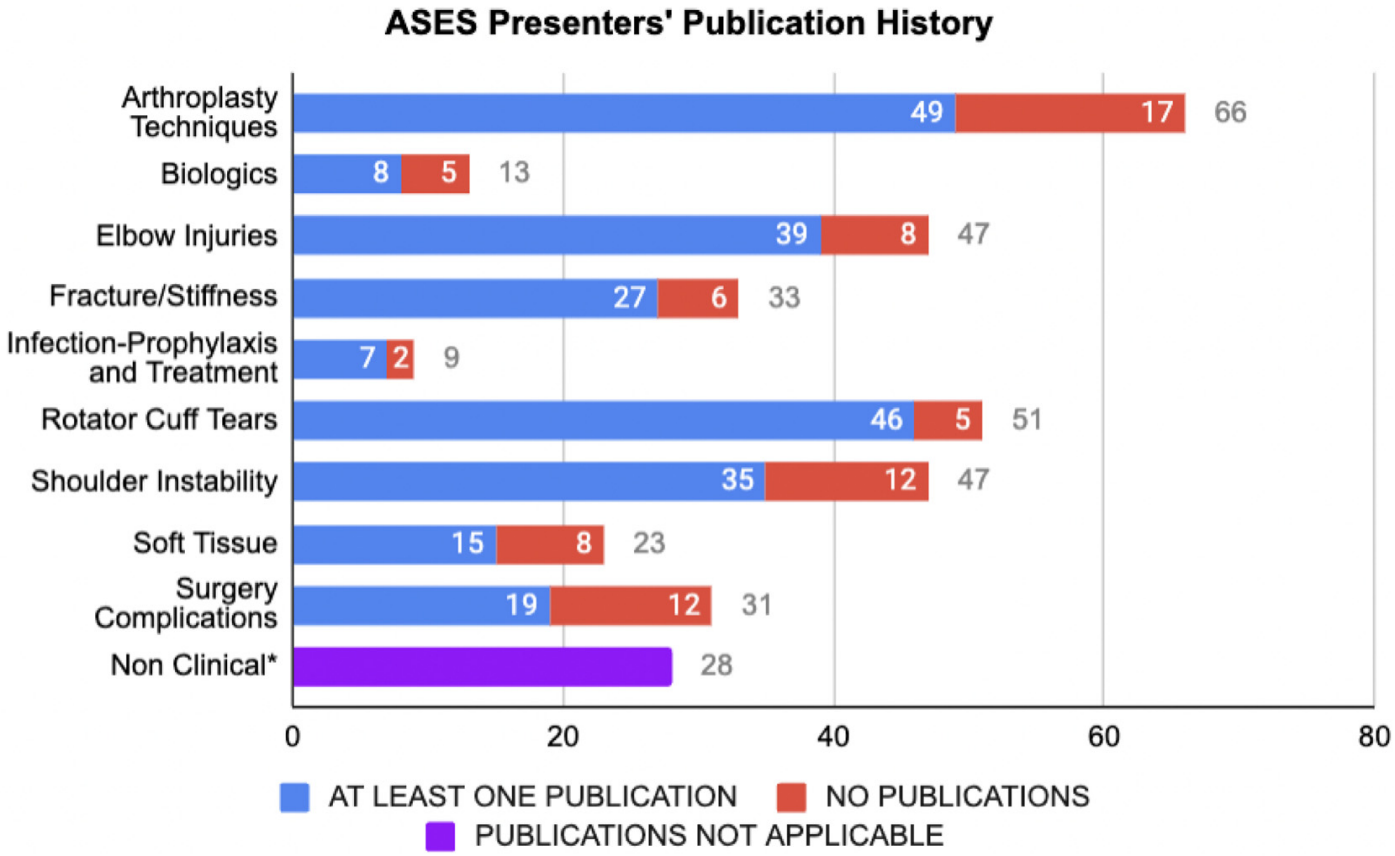
Presenters' publication history by ASES conference lecture categories. Arthroplasty techniques included shoulder and elbow arthroplasty. Biologics included cellular-based treatments. Elbow injuries include all injuries and associated procedures to the region excluding arthroplasty. Fractures/stiffness includes fractures of the shoulder region, humerus and forearm, as well as fracture sequelae. Surgical complications include, but are not limited to vascular injury, hardware complication, and bone loss. *∗*Variation in nonclinical discussions was too broad to categorize by publication history. *ASES*, American Shoulder and Elbow Surgeons.

Overall, discussions on RCT had the highest proportion of presenters with previous topic-specific publications (90.2%), followed by elbow injuries (83%) and fractures/stiffness (78.8%). In contrast, surgical complications had the lowest proportion of presenters with presenter topic-specific publications (61.3%), followed closely by biologics (61.5%; Fig. 1).

Lecturers had an average FWCI and H-index of 2.0 and 42.9, respectively. Lecturers who presented on infections held the highest average FWCI (2.55), while the lecturers discussing elbow injuries had the highest average H-index (59.6; Table III).

ICL instructors had an average FWCI and H-index of 1.5 and 33.4, respectively. ICL instructors who presented on biologics (only 1 event) had no previous topic-specific publications. Presentations that focused on RCTs had presenters with the highest average FWCI (4.7), while courses on biologics had the presenter with the highest H-index (58). Of note, there were no ICLs relating to infections-prophylaxis and treatment (Table IV).

**Table IV:** Academic metrics of instructional course lecturers.

Category	Number of ICLs	ICL lecturers with prior topic-specific publication	Average FWCI	Average H-index
Arthroplasty techniques	7	11/15 (73.3%)	2.0	44.3
Biologics	1	0/1 (0%)	0	58.0
Elbow injury	9	15/19 (78.9%)	1.3	43.7
Fracture/stiffness	5	6/8 (75.0%)	1.5	29.6
Infection	0	0	0	0.00
Rotator cuff tear	5	10/10 (100%)	4.7	37.0
Shoulder instability	7	12/17 (70.6%)	1.5	41.9
Soft tissue injury	6	9/12 (75.0%)	1.9	38.8
Surgical complications	4	1/6 (16.7%)	0.2	14.8
Nonclinical	6	-	-	25.5
Total	52	64/88 (72.7%)	1.5	33.5

*FWCI*, field-weighted citation impact; *H-index*, Hirsch index; *ICL*, instructional course lectures.

Symposium moderators had an average FWCI and H-index of 1.6 and 36.6, respectively. Moderators of discussions on infections held the highest average FWCI (2.8), while those discussing nonclinical topics had the highest average H-index (55; Table V).

**Table V:** Academic metrics of symposium moderators.

Category	Number of moderated discussions	Moderators with prior topic-specific publication	Average FWCI	Average H-index
Arthroplasty techniques	11	14/19 (73.7%)	2.0	35.3
Biologics	4	3/5 (60.0%)	0.7	54.7
Elbow injury	8	15/16 (93.8%)	1.5	29.1
Fracture/stiffness	9	14/17 (82.4%)	1.5	25.7
Infection	2	3/3 (100%)	2.8	42.3
Rotator cuff tear	8	12/13 (92.3%)	1.7	29.2
Shoulder instability	8	10/14 (71.4%)	1.8	19.5
Soft tissue injury	1	1/2 (50.0%)	0.4	28.5
Surgical complications	2	3/3 (100%)	1.9	47.3
Nonclinical	2	-	-	55.0
Total	56	75/92 (81.5%)	1.6	36.7

*FWCI*, field-weighted citation impact; *H-index*, Hirsch index.

## Discussion

Society annual meetings have been demonstrated to impact physician competency and improve patient clinical outcomes.^4^^,^^8^^,^^12^ Similar means of analysis have been used across other orthopedic subspecialty conferences to determine the expertise of presenters.^16^ This study employed similar methods to review the publication history and academic metrics of presenters at ASES meetings in an attempt to objectively evaluate the academic impact of selected presenters.

Presentations discussing surgical complications had the smallest proportion of presenters with previous topic-specific publications (61.3%). However, this may reflect the specific nature of the category. For example, individuals with extensive research in shoulder arthroplasty, may have fewer publications specifically related to complications of shoulder arthroplasty. Although the large fraction of presenters with previous topic-specific publications still reflects the adequate experience of invited speakers. In addition, the average H-Index for lecturers (44.3) and symposium moderators (47.3) of this topic was above the total average H-index in lecturers (42.9) and symposium moderators (36.6).

Presenters who discussed RCT had the largest proportion of previous topic-specific publications (90.2%). In addition, RCTs were the second most frequently presented topic across all of the conferences (36/226, 15.9%). This may reflect higher rates of diagnosis as well as increasing breadth of surgical treatment modalities for RCT over the past 20 years.^21^ A study on national trends in rotator cuff repair showed that between 1996 and 2006, there was a 141% increase in repair rates and a 600% rise in arthroscopic procedures coinciding with the rise in popularity of arthroscopic vs. open repairs as well as a shift from inpatient to outpatient surgeries.^7^ Consequently, associated RCT literature has also risen. Viswanath et al reported that 5017 studies regarding RCT were published between 2011 and 2020, a significant increase from the 1824 papers published between 2001 and 2010.^20^ Furthermore, newly developed strategies to address RCT likely contributed to the increasing number of RCT cases and associated research. Superior capsular reconstruction gained traction for management of massive, previously irreparable RCT in 2012, however, soon fell out of favor due to suboptimal functional outcomes.^6^^,^^10^^,^^19^ In addition, lower trapezius transfer became more popular for the treatment of massive irreparable RCT, specifically in the posterior-superior region, around 2009.^9^^,^^11^^,^^13^^,^^22^

In addition to previous topic-specific publications, academic metrics can be utilized as objective measures to determine the academic impact of presenters. The H-index is a number determined by calculating the authors' research productivity and overall academic impact based on their previous peer-reviewed publications.^15^^,^^14^ ASES presenters had an average H-index of 37.7, superior to the average H-index that was previously reported for shoulder and elbow surgery fellowship directors (24.2).^5^ The H-index of conference presenters demonstrates the advanced standard of productivity of ASES conference presenters.

Most presentations at ASES were given by physicians with at least 1 prior topic-specific publication (76.6%). For comparison, previous literature evaluating presenters at the American College of Foot and Ankle Surgeons and American Orthopedic Foot and Ankle Society reported that 29.2% of lecturers had at least 1 prior topic-specific publication.^16^ Currently, ASES has 1300 members, whereas American College of Foot and Ankle Surgeons and American Orthopedic Foot and Ankle Society societies have 7800 and 2500 members, respectively.^1^^,^^2^^,^^3^ The ability of ASES to provide such well published presenters with relatively lower number of members to select from demonstrates the strong academic focus amongst the ranks of the ASES.

This study was not without limitations. While our analysis utilized academic metrics in an attempt to define the academic qualifications of content presenters, authors acknowledge that there are other factors utilized by program planning committees in their selection of presenters for educational events. These factors may include the number of years in clinical practice, employment setting (academic vs. private practice), and previous public speaking experience. In addition, textbook chapters and other nonpeer reviewed literature were not considered in this study, although they are strong indicators of a presenter's subject expertise. The determination of content categories and subsequent categorization of presentations by content category was subjective. However, this was performed independently by two authors, with disputes determined by a third author to minimize risks of subjective bias. In addition, the categorization of presentations was limited as some speakers who had extensive research in broader areas, such as shoulder arthroplasty, may have fewer publications addressing the more specific topics in which they were invited to discuss, such as surgical complications. Although, the high proportion of presenters with previous topic-specific publications still demonstrates the expertise of invited speakers. H-indices and FWCIs were collected at present time, and may not represent the academic metrics at the time of conference presentation. In addition the average H-index of presenters was high, but there was a wide range of variability (range: 6-109) in H-indices. This range suggests that the average may not accurately reflect individual presenters. Also, lectures constituted 63.6% of all events. This was largely impacted by the 2017 and 2018 annual meetings which only included lecture presentations. The increased proportion of lecture presentations limited the ability to compare metrics between presenter roles. Finally, three content categories (infections, soft tissue injuries, biologics) consisted of less than ten total presentations, limiting the ability to draw definitive conclusions.

## Conclusion

A high percentage of ASES presenters had previous topic-specific publications as well as relatively high H-indices and FWCI. Speakers at ASES annual meetings are academically accomplished within the field of shoulder and elbow surgery.

## Disclaimers:

Funding: No funding was disclosed by the authors.

Conflicts of interest: The authors, their immediate families, and any research foundations with which they are affiliated have not received any financial payments or other benefits from any commercial entity related to the subject of this article.

## References

[1] American College of Foot and Ankle Surgeons What is AFCAS?. https://www.foothealthfacts.org/what-is-acfas#:%7E:text=What%20is%20ACFAS%3F.

[2] American Orthopaedic Foot and Ankle Society Apply for AOFAS membership. https://www.aofas.org/membership/apply#:%7E:text=Apply%20for%20AOFAS%20Membership.

[3] American Shoulder and Elbow Surgeons About ASES. https://www.ases-assn.org/about-ases/#:%7E:text=Through%20continuing%20medical%20education%2C%20the,consists%20of%20over%201300%20members.

[4] Cervero R.M., Gaines J.K. (2015). The impact of CME on physician performance and patient health outcomes: an updated synthesis of systematic reviews. J Contin Educ Health Prof.

[5] Chopra A., Wright M.A., Klifto C.S., Anakwenze O., Murthi A.M. (2022). Leadership trends in shoulder and elbow surgery fellowship directors: a cross-sectional study. J Am Acad Orthop Surg Glob Res Rev.

[6] Chung S.W., Kim D.H., Lee H.J., Hong W.K., Chung S.H., Yoon J.P. (2023). Superior capsular reconstruction for irreparable rotator cuff tear: a review of current methods. Clin Shoulder Elb.

[7] Colvin A.C., Egorova N., Harrison A.K., Moskowitz A., Flatow E.L. (2012). National trends in rotator cuff repair. J Bone Joint Surg Am.

[8] Davis D., O'Brien M.A., Freemantle N., Wolf F.M., Mazmanian P., Taylor-Vaisey A. (1999). Impact of formal continuing medical education: do conferences, workshops, rounds, and other traditional continuing education activities change physician behavior or health care outcomes?. JAMA.

[9] Desai V., Stambulic T., Daneshvar P., Bicknell R.T. (2022). Lower trapezius tendon transfer for irreparable rotator cuff injuries: a scoping review. JSES Rev Rep Tech.

[10] Dimock R.A.C., Malik S., Consigliere P., Imam M.A., Narvani A.A. (2019). Superior capsule reconstruction: what do we know?. Arch Bone Jt Surg.

[11] Elhassan B.T., Wagner E.R., Werthel J.D. (2016). Outcome of lower trapezius transfer to reconstruct massive irreparable posterior-superior rotator cuff tear. J Shoulder Elbow Surg.

[12] Forsetlund L., Bjørndal A., Rashidian A., Jamtvedt G., O’Brien M., Wolf F. (2009). Continuing education meetings and workshops: effects on professional practice and health care outcomes. Cochrane Database Syst Rev.

[13] Ghoraishian M., Stone M.A., Elhassan B., Abboud J., Namdari S. (2020). Techniques for lower trapezius tendon transfer for the management of irreparable posterosuperior rotator cuff tears. J Orthop.

[14] Hirsch J.E. (2007). Does the H index have predictive power?. Proc Natl Acad Sci U S A.

[15] Hirsch J.E. (2005). An index to quantify an individual's scientific research output. Proc Natl Acad Sci U S A.

[16] Hyer C.F., Casciato D.J., Rushing C.J., Schuberth J.M. (2022). Incidence of scholarly publication by selected content experts presenting at national society foot and ankle meetings from 2016 to 2020. J Foot Ankle Surg.

[17] Kay J., Memon M., de Sa D., Simunovic N., Athwal G., Bedi A. (2016). Level of clinical evidence presented at the open and closed American Shoulder and Elbow Surgeons annual meeting over 10 years (2005–2014). BMC Musculoskelet Disord.

[18] Kearney P., Simoons M., Ryden L., Kirchhof P., Pries A., O’Morain C. (2019). The medical profession, industry, and continuing medical education: finding the balance that's right for patients. Am J Med.

[19] Makovicka J.L., Chung A.S., Patel K.A., Deckey D.G., Hassebrock J.D., Tokish J.M. (2020). Superior capsule reconstruction for irreparable rotator cuff tears: a systematic review of biomechanical and clinical outcomes by graft type. J Shoulder Elbow Surg.

[20] Viswanath A., Monga P. (2021). Trends in rotator cuff surgery: research through the decades. J Clin Orthop Trauma.

[21] Yanik E.L., Chamberlain A.M., Keener J.D. (2021). Trends in rotator cuff repair rates and comorbidity burden among commercially insured patients younger than the age of 65 years, United States 2007-2016. JSES Rev Rep Tech.

[22] Ye L., Han D., Zhang Q., Yang X., Tung T.H., Zhou X. (2022). Early efficacy assessment of arthroscopic lower trapezius transfer with tendon autograft in the management of massive irreparable posterosuperior rotator cuff tears. Front Surg.

